# Surgical difficulties for Total Knee Replacement in Stickler syndrome: A case report

**DOI:** 10.1186/1757-1626-1-179

**Published:** 2008-09-24

**Authors:** Gopalkrishna G Verma, Adel Zarough, KH Suraliwala

**Affiliations:** 1 Southport and Ormskirk District General Hospital, Town lane, Kew, Southport. PR8 6PN, UK

## Abstract

Stickler syndrome is believed to be the most common connective tissue disorder in Europe and the USA. Severe osteoarthritis sets in at very early age in 3^rd ^to 4^th ^decade of life necessitating joint arthroplasty. This case report highlights the intraoperative surgical difficulties faced by the surgeon and the planning needed for the operation.

## Introduction

Stickler syndrome was described Dr Gunnar Stickler in 1960 at the Mayo Foundation in Minnesota, USA. Stickler syndrome is an autosomal dominant group of hereditary conditions characterized by findings of myopia, cataract, and retinal detachment; hearing loss that is both conductive and sensorineural; midfacial underdevelopment and cleft palate (either alone or as part of the Robin sequence); and mild spondyloepiphyseal dysplasia and/or precocious arthritis. Variable phenotypic expression of Stickler syndrome occurs both within families and among families; interfamilial variability is in part explained by locus and allelic heterogeneity. 1 in 10,000 people may be affected by Stickler syndrome. [1-5] The diagnosis of Stickler syndrome is clinically based. At present, no consensus on minimal clinical diagnostic criteria exist. [4]

Mutations affect at least 4 genes which control and direct collagen synthesis. Four types of Stickler syndrome are known which are COL2A1, COL11A1, COL112A, unknown gene. [1,5] The diagnosis can be confirmed by molecular genetic testing; however, it is primarily used to obtain information for genetic counselling. Affected individuals have a 50% chance of passing on the mutant gene to each offspring. [4] The degree of joint involvement within Stickler Syndrome is extremely variable. The most common affected joints are the hips, knees, ankles, neck, back, hands and feet.

Osteoarthritis sets in as early as in 3^rd ^and the 4^th ^decade. Hypermobile joints tend to stiffen as osteoarthritis sets in.

There is a lack of information in the literature on surgical difficulties faced during total knee replacement (TKR) in Stickler syndrome. This is the first attempt to describe the surgical findings and difficulties encountered during the total knee replacement surgery.

## Case report

We present a case of Stickler syndrome in a 52 year old female who had early wide spread osteoarthritis, macular degeneration and deafness. Her mother had all the above features along with cleft palate. Patient's daughter was the third generation to also have Sticklers syndrome in the family. Patient suffered from severe osteoarthritis of both the knees.

**Figure 1:**
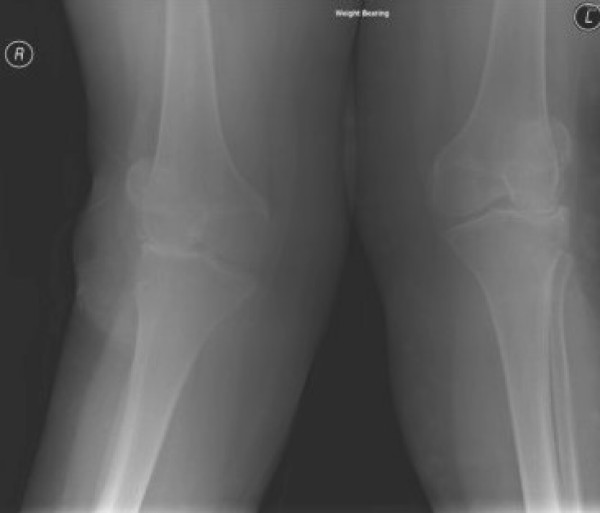
**Preoperative weight bearing x-ray of both the knees**. Showing severe tricompartmental arthritis and valgus deformity of the right knee.

**Figure 2:**
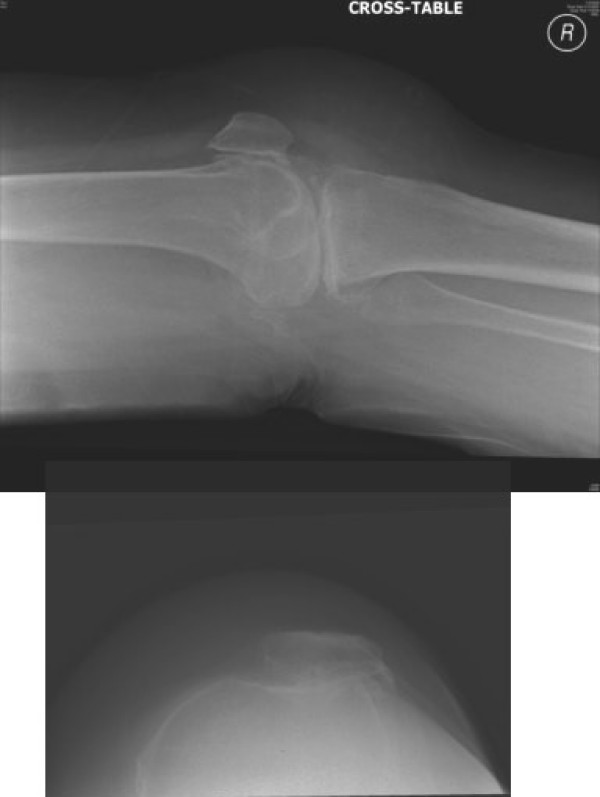
**Preoperative lateral x-ray of the right knee and skyline of the patella**. Showing severe arthritis and maltracking of the right patella.

The patient had severe valgus deformity 18° with patellar maltracking. (Figure 1 &2) Intraoperatively there were severe osteoarthritic changes with large areas of exposed subchondral bone in both the femoral and tibial condyles. The bone was very soft. The knee ligaments were very lax. This caused uneven flexion and extension gaps causing difficulties with ligament balancing. There was gross instability in flexion due to larger flexion gap which was corrected by inserting a thicker tibial component, & the resultant loss of extension was then corrected by recutting distal femur more proximally. Thick tibia polyethylene insert of size 15 mm was accepted to balance the knee.

Tissue samples of the bone and synovium were sent for histology. Both the femoral and tibial tissue showed fibrillation and erosion of the articular cartilage with exposure of the subcondral bone. Attempted fibrocartilaginous repair was overwhelmed by the destructive process. Osteophytes surrounded the edge of the articular surfaces. The subcondral bone showed focal remodelling. The bone had a normal lamellar structure. The marrow tissue was mainly fatty with occasional small foci of normal haemopoietic tissue. The appearances were those of primary and secondary osteoarthritis, with no evidence of a disorder of the bone structure. The synovium showed mild reactive changes with papillary architecture, normal intimal layer and mild, patchy lymphocytic infiltrate, the changes consistent with degenerative joint disease.

## Discussion

Hypermobile joints can be due to following four causes, shallow joints, abnormalities of collagen, abnormal tone of the muscles, abnormalities with the kinaesthetic sensation. Problems with collagen proteins will cause weak or stretched ligaments. The tone of the muscles will affect how the joints are held loosely or rigidly. Early onset of osteoarthritis leaves these patients needing total knee replacement at young age.

**Figure 3:**
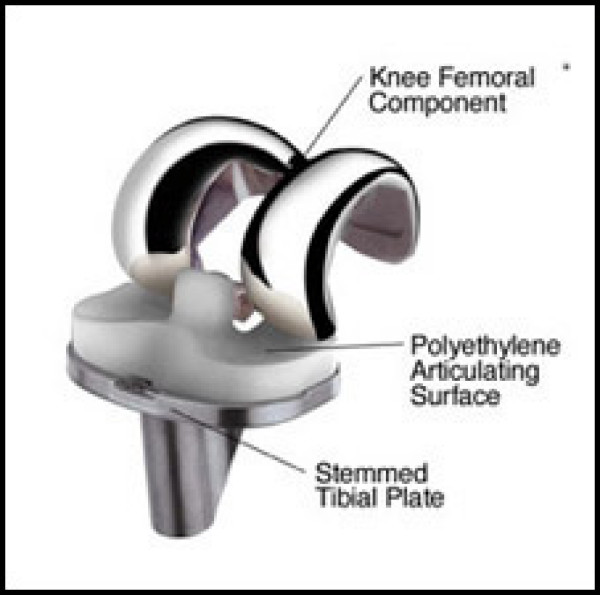
Total Knee Replacement implant.

A TKR replaces your diseased knee joint with an artificial knee joint (Figure 3) and eliminates the damaged bearing surfaces that cause pain. The lower part of the replacement knee joint is comprised of a flat metal plate and stem that is implanted in the tibial bone. Next, a polyethylene insert is clipped into the tibial tray to serve as the new knee bearing surface. The upper part of the replacement knee joint consists of a contoured metal shield that fits around the lower end of the femur. The outer surface of the contoured metal shield is shaped to allow the patella to slide up and down in its groove.

Precision jigs/guides are used to help make sure the cuts are made at the correct angles so the bones will align properly after the implants are fixed. Flexion gap is the space between the posterior coronal cut on the distal femur & transverse cut on proximal tibia, while knee is in flexion. Extension gap is the space between transverse cut on distal femur & transverse proximal tibial cut while the knee is in complete extension.

In TKR surgery soft tissue balancing is half the procedure and the other half is the precision of the bone cuts. Soft tissue balancing during total knee replacement (TKR) comes with a unique set of problems – it is a subjective art that is hard to master and even more difficult to teach. [6] Abnormalities of collagen synthesis causing stretched ligaments, affection with the muscle tone, hypermobility and variable early onset osteoarthritis presents an unique set of problems which are different from the routine TKR operation as indicated for elderly osteoarthritis of the knee. Careful preoperative planning will help anticipate these problems and deal them successfully.

Anaesthetics risks are due to microganthia and cleft palate causing airway difficulties and mitral valve prolapse causing cardiac abnormality. [3,7] Patients need to be counselled preoperatively before they undergo major surgery. [4] Longitivity of the prosthesis in these patients is still unknown, hence patients needs to understand the prospect of multiple revision surgeries in their life time.

## Conclusion

Stickler's syndrome demands extra attention and cautious approach when you consider major orthopaedic procedure like total knee replacement. Knee ligament balancing can be huge problem. Careful preoperative planning and precise surgical technique will help overcome unexpected intra-operative difficulties and achieve a successful outcome.

## Abbreviations

TKR: Total knee replacement.

## Competing interests

The authors declare that they have no competing interests.

## Authors' contributions

GV conceived the idea, did the literature search, followed up the reports and the patient, wrote the case report and finalised and approved the copy. Author AZ helped with the literature search, followed up the patient and read and approved the final copy. Author SK followed up the patient, coordinated the case report, approved and finalised the copy.

## Consent

Written informed consent was obtained from the patient for publication of this case report and accompanying images. A copy of the written consent is available for review by the Editor-in-Chief of this journal.
